# Numerical simulation of size effect of defective rock under compression condition

**DOI:** 10.1038/s41598-023-27651-y

**Published:** 2023-01-09

**Authors:** Zeyu Hu, Liangfu Xie, Yongjun Qin, Xuejun Liu, Jiangu Qian

**Affiliations:** 1 College of Civil Engineering and Architecture, Xinjiang University, Urumqi, 830046 China; 2 Xinjiang Civil Engineering Technology Research Center, Urumqi, 830046 China; 3 Xinjiang Academy of Architectural Science (Limited Liability Company), Urumqi, 830054 China; 4 Department of Geotechnical Engineering, Tongji University, Shanghai, 200092 China

**Keywords:** Engineering, Physics

## Abstract

The existence of various types of damage, small cracks, some large voids and the size of the sample in the rock will make the experimental results show great discreteness. In this paper, based on the results of laboratory experiments, a numerical model of large flawed rock samples is established by using particle flow software PFC2D, and the mechanical response of rocks with different length-diameter ratios and different flaw positions in uniaxial compression experiments is discussed. The results show that the specimen size has a significant effect on the crack characteristics, mechanical characteristics and energy characteristics of rock mass. From the perspective of energy and crack characteristics, the total number of cracks after the failure of the defective rock sample is slightly lower than that of the intact rock sample, resulting in a slightly lower peak strain energy during the rock failure process. From the mechanical properties of rock samples, the Poisson’s ratio of intact rock samples is slightly smaller than that of defective rock samples. The strength of the defective sample is weakened relative to the complete rock sample, and the relationship formula between the weakening range and the aspect ratio is obtained through analysis. Moreover, different defect locations lead to different crack processes and crack modes, resulting in different uniaxial compressive strength.

## Introduction

In engineering practice, the problem of how to determine the physical and mechanical parameters of rock mass is always unavoidable. However, during the experiment, because the rock is a heterogeneous material, there are various types of damage, small cracks and some large voids inside it. And the physical and mechanical properties of rock samples with different sizes are obviously different, that is, the size effect of rock. This will make the reduction coefficient different when selecting the laboratory mechanical parameters for rock deformation analysis, which will lead to a large error in the prediction of rock mass deformation. Therefore, the size effect of rock and the variability of rock mass must be considered when selecting and applying the physical and mechanical properties of rock. For this reason, domestic and foreign scholars have done a lot of physical experiments^1–5^, numerical simulation experiments^6–8^ and theoretical research^9–11^, and gradually have a deep understanding of this problem.

In the study of discreteness of rock experimental results, it is considered that the reason why the experimental results of the same rock sample show discreteness is that the rock is a complex natural structure with various tiny defects inside it^12–14^. The study of rock size effect is mostly from two aspects, one is from the experimental instruments and experimental methods. During the experiment, the contact between the pressure plate of the experimental instrument and the rock sample is not completely smooth. As the experiment proceeds, a large friction will be generated on the contact surface, which will have a more obvious impact on the results of the experiment, that is, the end effect in the process of rock experiment^15,16^. Sun et al.^17^ simulated the uniaxial compression test of rock without end friction by numerical simulation. The results show that the size effect of rock compressive strength is controlled by end constraints. Xu, Y.H et al.^18^ studied the influence of end effect on rock strength in triaxial tests. The results show that the end effect has a significant effect on rock strength. Yota Togashi et al.^19^ conducted triaxial tests on tuff under different end constraints, and found that the direction of shear strain and principal strain of the specimen was significantly affected by the end constraints.

For another is from the structural characteristics of rock research. Rock is a complex natural structure. Due to the long-term geological structure and environmental impact during and after the formation of rock, there are often various types of damage, small cracks and some large voids in the rock. These defects have a significant impact on the physical and mechanical properties of rock^20–23^. Hu Gaojian and Ma Gang^24^ studied the relationship between the parallel joint spacing and the size effect of rock samples. They concluded that the joint distribution of rock samples has an exponential relationship with the size effect of rock uniaxial compressive strength. Yota TGuan Rong et al.^25^ studied the size effect of marble under different damage conditions by heat treatment, and found the relationship between the peak strength of marble and temperature and sample size. Zhu Jianbo et al.^26^ compared the stress wave attenuation amplitude of rocks with different damage degrees, and concluded the relationship between stress wave attenuation and initial damage degree of rocks. Ma Junbiao et al.^27^ carried out uniaxial compression test on composite defect rock samples with double holes and double cracks by DEM method. They concluded that the defects of rock specimens had a significant effect on their strength characteristics. Hou Rongbin et al.^28^ conducted multi-load creep tests on sandstones with different initial damage levels. They found that the initial damage has a significant effect on the creep behavior of rock and proposed a new nonlinear creep damage model to predict the creep behavior of sandstone under different initial damage states. Wang Yongqian et al.^29^ studied the static and dynamic characteristics of limestone with different initial damage degrees by using longitudinal wave velocity and static-dynamic compression test. They found that strain rate and initial damage have obvious influence on rock compression characteristics. Wen Lei et al.^30^ compared different methods for calculating the initial damage degree, and found that when the initial damage is small, the difference between different calculation methods is small, and when the damage degree is heavy, the difference is obvious. Liang, Z et al.^31^ studied the change of mechanical behavior after rock damage caused by pre-peak unloading. It is found that a micro fracture in the rock is transformed from brittle–ductile damage, while macroscopic damage occurs in the form of a “shear”-“splitting”-“mixed shear-splitting” damage process. Taqi Alzaki et al.^32^ studied the influence of structural heterogeneity on rock failure by numerical simulation. The results show that microscopic heterogeneity plays an important role in controlling microscopic mechanical behavior and macroscopic response under uniaxial compression loading.

The size effect of rock and the variability of experimental results are mainly concentrated in the above aspects. Most of the research on the defects of rock focuses on the evaluation of the damage degree of rock samples and the description of the damage process. It is difficult to intuitively reflect the change of physical and mechanical properties before and after rock damage and the change rule after re-failure. The study of rock size effect is also biased towards intact rock. In order to supplement the lack of research on defective rock samples and provide a basis for the selection of mechanical parameters of rock, a numerical model of rock samples with large defects is established in this paper, and the variation law of mechanical properties and the size effect of rock samples with large defects are analyzed from multiple angles. The conclusions can provide reference for the variability of rock experimental parameters and the size effect of rock.

## Validation of DEM numerical simulation

### Selection of model parameters

The two-dimensional particle flow code (PFC2D) is a discrete element program that represents solid materials as a set of circular particles. In PFC2D the specimen consists of a plurality of particles and the particles are bonded together by assigning contact rules to the contact between the particles. The physical and mechanical properties of the specimen cannot be set directly, but are controlled by the geometric and physical parameters of the particles and the contact rules. Therefore, the microscopic parameters required in PFC2D are based on the macroscopic parameters obtained in physical experiments, and then obtained after repeated tests in PFC2D software by simulating rock compression and other experiments^33,34^. Therefore, it is particularly important to choose a reasonable contact model. There are many embedded contact models in PFC2D, which includes Linear Contact Bond Model, Parallel Bond Model, Soft-Bond Model, Rolling Resistance Linear Model etc. Among the above constitutive models, the main advantage of Parallel Bond Model is that it can replace the complex empirical constitutive behavior with simple particle contact logic, which can well simulate the mechanical behavior of rock.

**Figure 1:**
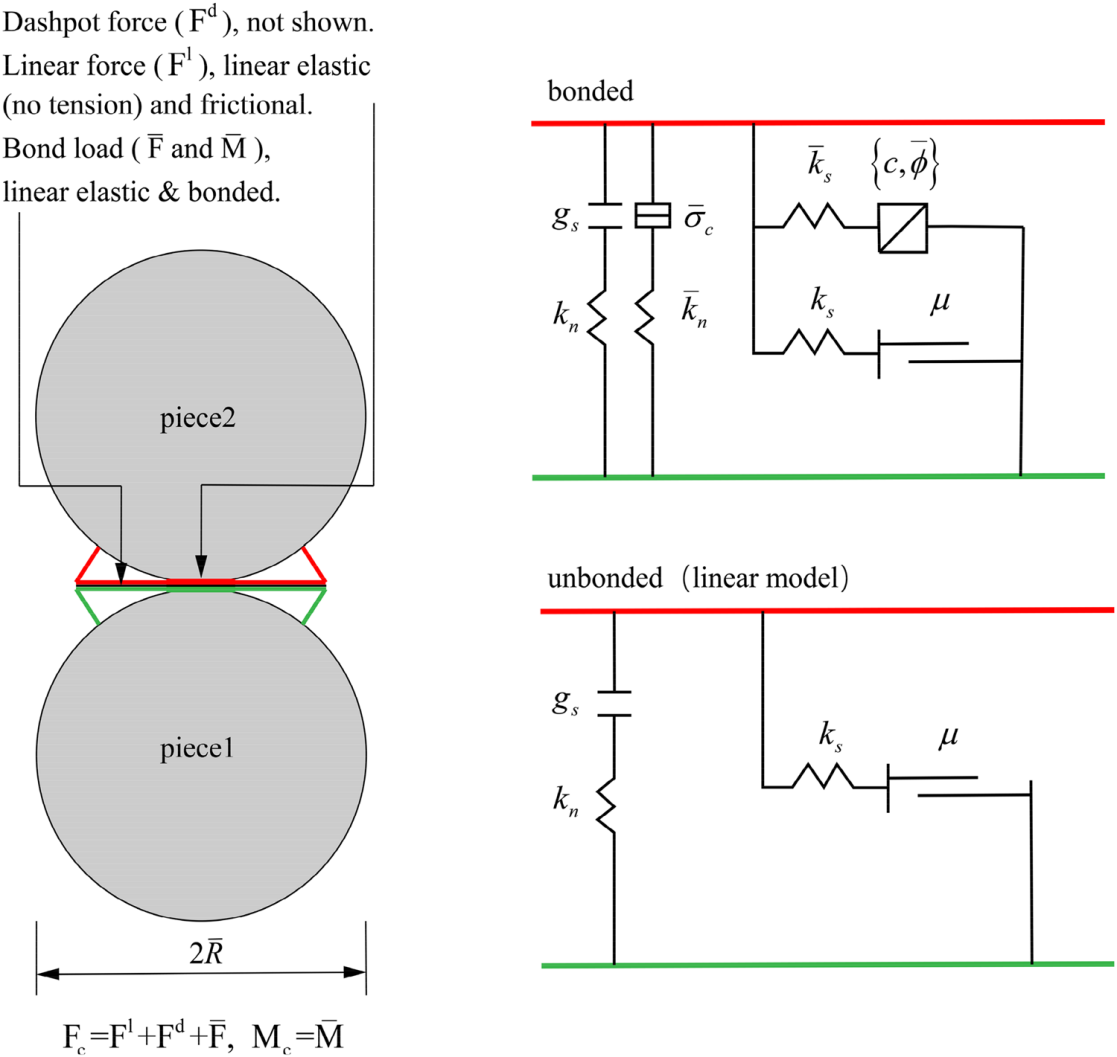
Behavior and rheological components of parallel bond model^35^.

As shown in Fig. 1, there are two interface behaviors in the parallel bond model : The first interface is equivalent to a linear model, which does not resist relative rotation and controls slip by applying a Coulomb limit to the shear force. The second interface is a parallel bond. When it is bonded, it resists relative rotation and its behavior is linear elasticity. When it is subjected to a load exceeding the strength limit, the bond will break and not bear the load.

Where ${\mathrm{F}}_{\mathrm{c}}$ is the force between particles, ${\mathrm{M}}_{\mathrm{c}}$ is the moment between particles, ${\mathrm{F}}^{\mathrm{l}}$ is the linear force, ${\mathrm{F}}^{\mathrm{d}}$ is the dashpot force, $\overline{\mathrm{F} }$ is the parallel-bond force, $\overline{\mathrm{M} }$ is the parallel-bond moment. ${\mathrm{g}}_{\mathrm{S}}$ is the cumulative relative normal displacement of the piece surfaces, $\mu$ is the friction coefficient, ${k}_{n}$ and ${\overline{k} }_{n}$ is the normal stiffness, ${k}_{s}$ and ${\overline{k} }_{S}$ is the shear stiffness, ${\tilde{\sigma }}_{c}$ is the tensile strength, *c* and $\overline{\phi }$ is the Cohesion and Friction angle.

**Figure 2:**
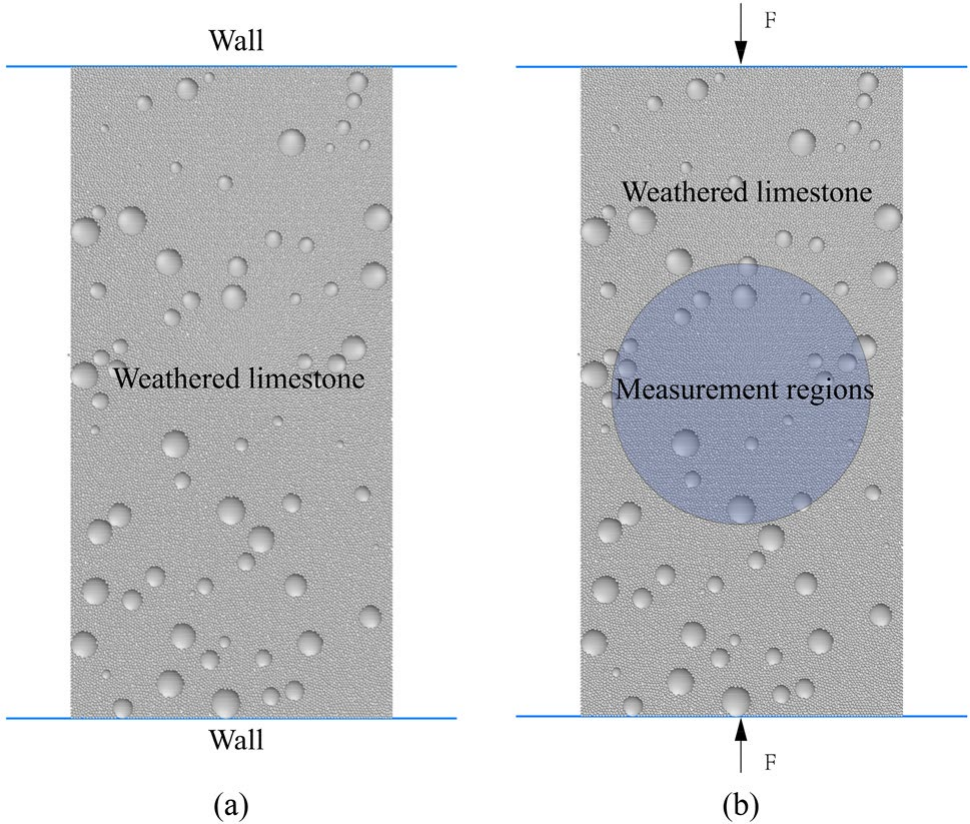
Model diagram (**a**) Initial diagram of model (**b**) Model loading and monitoring diagram.

In this study, the parallel bond model is used to establish the numerical model of uniaxial compression experiment as shown in Fig. 2a. The initial model size is φ50mm × 100 mm and consists of approximately 30,000 particles. The pressure is applied to the sample by setting the wall in the upper and lower parts of the model and controlling the displacement of the wall. The measuring circle and related monitoring commands are set inside the sample to monitor the changes of stress and strain inside the sample (Fig. 2b).

In this paper, slightly weathered limestone as the research object, through numerical simulation method to simulate uniaxial compression experiment. In order to verify the accuracy of the numerical model of rock sample, this paper adjusts the microscopic parameters of the model by trial-and-error method and experimental experience value^36,37^ according to the macroscopic parameters of rock mass obtained by field physical experiment. A more reasonable model microscopic parameters are finally obtained, as shown in Table 1.

**Table 1:** Microscopic parameters of the model.

Model scale/(mm)	Maximum particle radius/(mm)	Minimum particle radius/(mm)	Bond cohesion/(MPa)	Bond tensile strength/(MPa)	Friction coefficient	Bond effective modulus/(Gpa)	Bond stiffness ratio
50 × 100	2.5	0.2	56	56	0.2	3.20	2.2

**Figure 3:**
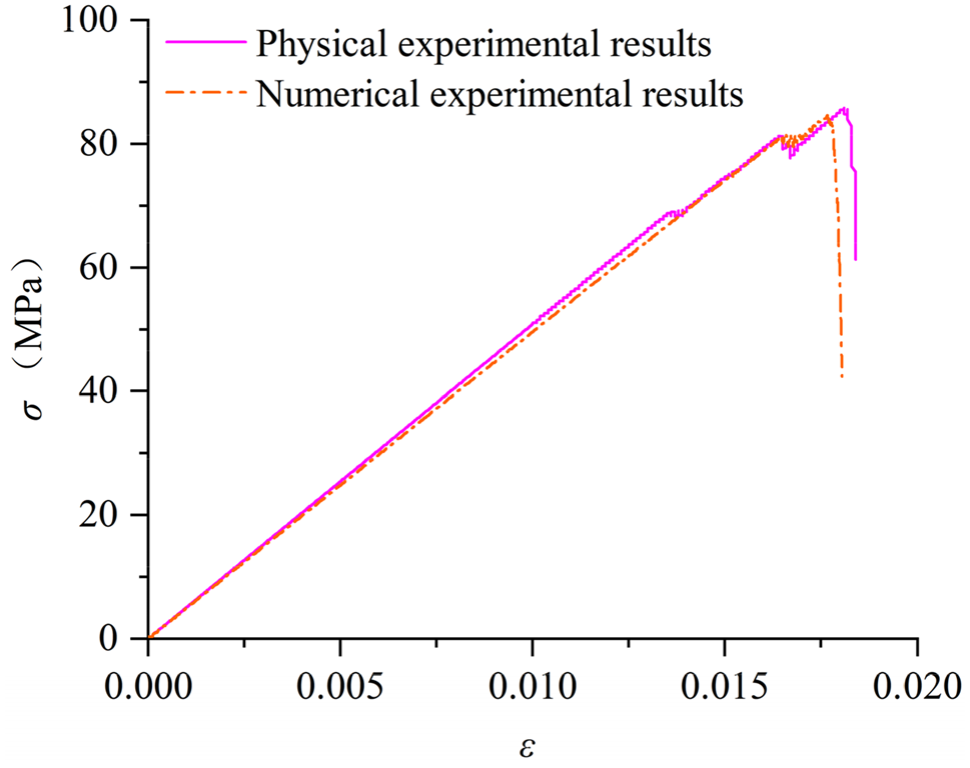
Stress and strain curves of physical experiments and numerical simulation experiments.

Based on the above microscopic parameters, the stress–strain curve comparison diagram (Fig. 3) and macroscopic parameter error table (Table 2) of rock samples are obtained by numerical simulation.

**Table 2:** Rock macroscopic parameter deviation table.

Rock types	Experimental method	Elastic modulus (Gpa)	Poisson’s ratio	Uniaxial strength(Mpa)
Weathered limestone	Physics experiment	5.0	0.30	85.921
	PFC	4.96	0.303	84.616
	Deviation /%	0.80	1.0	1.52

It can be seen from the data in the table that the macroscopic physical and mechanical parameters of rock samples obtained by numerical simulation based on the above microscopic parameters are basically consistent with the results obtained in physical experiments, and the maximum error is 1.52%, which proves the rationality of the numerical model used in this paper.

### Verifying the model size effect

**Figure 4:**
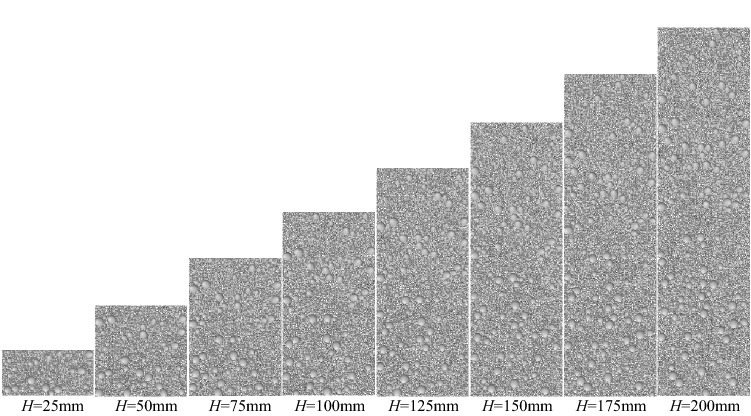
Schematic diagram of rock samples with different aspect ratios.

**Figure 5:**
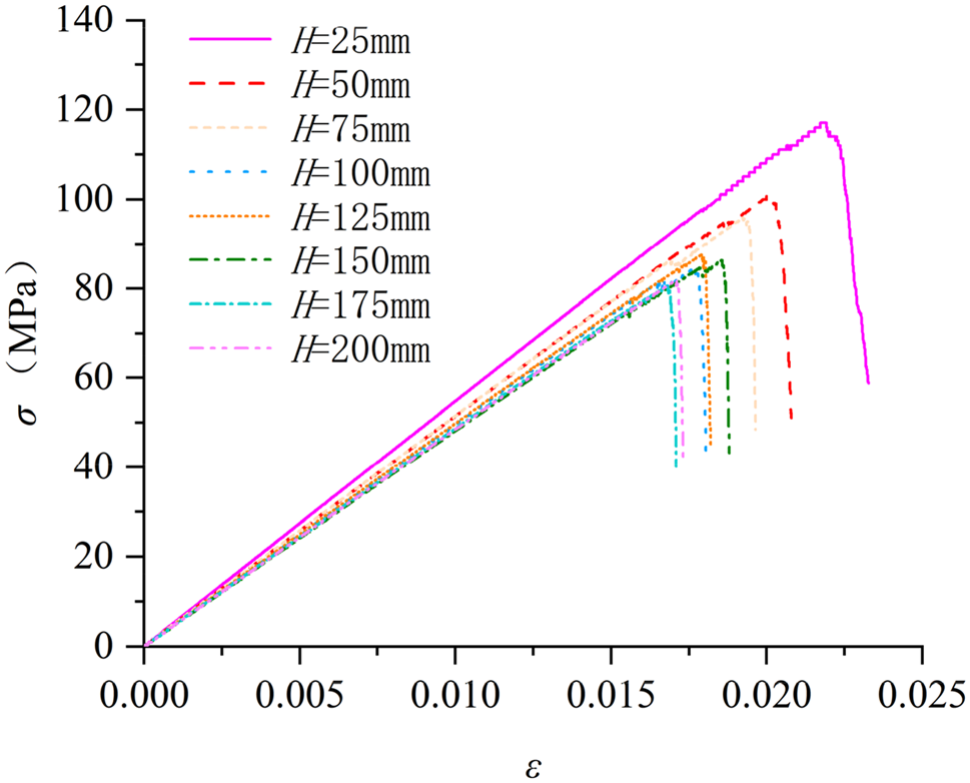
Intact rock stress–strain curve.

In this paper, samples with different aspect ratios were obtained by changing the height (H) of rock samples by fixing the diameter of rock samples (D = 50 mm) (The model diagram is shown in Fig. 4). Then a uniaxial compression test is performed on each specimen, and the stress–strain results of the experimental rock specimens are shown in Fig. 5 (σ is uniaxial compressive strength of sample). The stress–strain curves of the weathered limestone described in Fig. 5 show that the specimens exhibit brittle failure. When H = 25 mm (aspect ratio = 0.5), the peak stress and maximum strain are the maximum values of all experimental results. When H > 75 mm (aspect ratio > 1.5), the stress–strain curve of the sample begins to stabilize within a certain range. It can be seen from Fig. 4 that with the increase of the aspect ratio of the sample, the uniaxial compressive strength of the rock sample shows an overall decreasing trend, and the change is particularly significant before H/D = 2.0. Between H/D = 0.5 and H/D = 2.0, the uniaxial compressive strength of the rock sample decreased from 117.192 MPa to 84.6161 MPa, a decrease of 32.58 MPa. After H/D = 2.0, the change of uniaxial compressive strength of rock sample tends to be gentle and fluctuates within a certain range.

At present, a large number of scholars have studied the size effect of rock compressive strength. And many empirical formulas have been obtained based on a large number of experimental data. For example: Some scholars proposed an empirical formula based on a large number of physical experimental data:

1 $$C_{C} = \frac{{C_{P} }}{{0.778 - 0.222\frac{D}{h}}},$$

where ${C}_{P}$ is the uniaxial strength of rock specimen with height larger than diameter or transverse dimension, ${C}_{C}$ is the uniaxial compressive strength of an equivalent cubic rock specimen, *h* is the length of rock sample, *D* is the diameter of rock sample.

Liu Baochen et al.^1^ proposed an improved formula based on a large number of experimental data at home and abroad:

2 $$\sigma_{C} = \gamma_{C} + \alpha_{C} \exp \left( { - \beta_{C} D} \right),$$

where *D* is the side length or diameter of the stress section, ${\sigma }_{C}$ is the uniaxial compressive strength of the rock sample, ${\gamma }_{C}, {\alpha }_{C}, {\beta }_{C}$ are the characteristic parameters of the material.

Jing Hong Wen et al.^6^ proposed an attenuation formula for the uniaxial compressive strength of rock:

3 $$\Delta \sigma = \Delta \sigma_{0} \left( {a + b/\lambda } \right),$$

where $\lambda$ is the aspect ratio of the cylindrical specimen rock sample, $a$ and $b$ are material parameters, $\Delta {\sigma }_{0}$ is the reference value of rock strength attenuation, $\Delta \sigma$ is the amplitude of rock strength attenuation with different aspect ratio.

Wu et al.^38^ conducted uniaxial experiments on green sandstone and basalt, and proposed that the size effect of rock samples should be reflected in the form of piecewise functions. The fitting formula of green sandstone is:

4 $$R_{C} = \left\{ \begin{gathered} 1.07^{D} + 11.00 \hfill \\ 4.46\ln D - 20.48 \hfill \\ \end{gathered} \right.\begin{array}{*{20}c} {} \\ {} \\ \end{array} \begin{array}{*{20}c} {D = 20 \sim 50\,mm} \\ {D = 50 \sim 100\,mm} \\ \end{array} ,$$

where ${R}_{C}$ is the uniaxial strength, *D* is the diameter of the specimen.

However, most of the above scholars study the size effect of rock by controlling the diameter of the sample, and the consideration of the length of the sample is insufficient. Therefore, this paper studies the size effect of rock by fixing the sample diameter and changing the sample length. In this paper, the experimental results of the numerical model are compared with the empirical formula (Formula (5)) proposed by Yang Shengqi et al.^9^.

5 $$F_{0} = F_{2} \exp \left[ {a + b/\left( {L/D} \right)} \right],$$

where ${F}_{0}$ is the compressive strength of rock specimen with any length-diameter ratio under uniaxial compression, ${F}_{2}$ is the compressive strength of standard rock specimen, $L$ is the length of cylindrical specimen, $D$ is the radius of cylindrical specimen, *a* and $b$ are material parameters.

**Figure 6:**
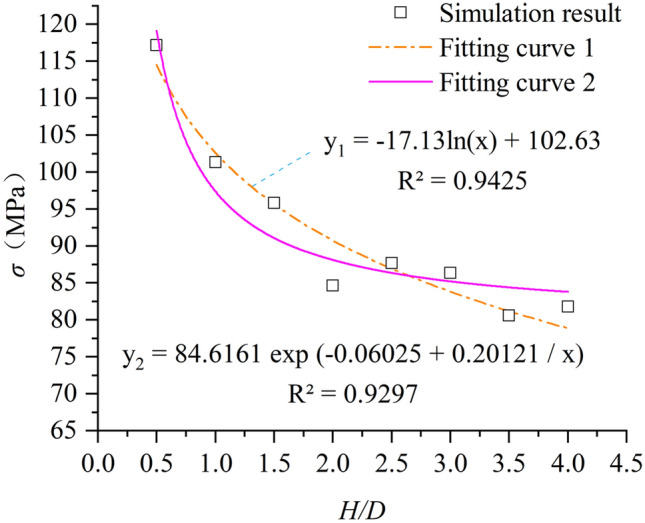
Size effect of compressive strength of intact rock sample.

Figure 6 shows that the numerical simulation results are in good agreement with the research conclusions (fitting curve 2) obtained by Yang Shengqi et al.^9^, which proves the rationality of the experimental parameters used in this paper.

## Design and results of uniaxial compressive experiment for defective rock mass

In practical engineering, the probability of large defects in large-sized rock is much higher than that of small-sized rock samples used in laboratory experiments. And it is not guaranteed that the experimental results of smaller rock specimens can accurately reflect the physical and mechanical properties of larger rock specimens. Based on this, this paper carries out the uniaxial compression experiment of rock with defects.

### Specimen morphology

Using the PFC2D microscopic parameters obtained from the above calibration, eight sets of rock sample models with a diameter of 50 mm and a aspect ratio of 0.5 to 4.0 were established. And a defect with a diameter of d = 5.0 mm is generated in the model.

According to the aspect ratio of the sample, it can be divided into 8 groups. There are 9 samples in each group, which are the combination of the position of the sample defect in the middle of the model (x1 = 24.999 mm ), the left side (x1 = 8.333 mm), the right side (x1 = 41.667 mm) and the upper part (y1 = 0.75H), the middle part (y1 = 0.5H) and the lower part (y1 = 0.25H). The specific distribution of model size and defect location is shown in Table 3.

**Table 3:** Distribution of sample defects.

Aspect ratio	Model scale		Model diagram
	*H* (mm)	*D* (mm)	
0.5	25	50	
1	50	50	
1.5	75	50	
2	100	50	
2.5	125	50	
3	150	50	
3.5	175	50	
4	200	50	

### Experimental result

Table 4 shows the uniaxial compressive strength values of the defective specimens obtained by monitoring the stress–strain curves of the specimens during the test in the above 8 groups of 72 specimens.

**Table 4:** Uniaxial compressive strength of defective rock samples.

Cases	*H*/*D*	Uniaxial compressive strength at different defect positions (MPa)								
		Defect position (*x*_1_, *y*_1_)								
		*x*_1_ = 8.33 mm			*x*_1_ = 24.999 mm			*x*_1_ = 41.667 mm		
		*y*_1_ = 3/4*H*	*y*_1_ = 1/2*H*	*y*_1_ = 1/4*H*	*y*_1_ = 3/4*H*	*y*_1_ = 1/2*H*	*y*_1_ = 1/4*H*	*y*_1_ = 3/4*H*	*y*_1_ = 1/2*H*	*y*_1_ = 1/4*H*
s1	0.5	98.873	105.296	99.9808	100.817	101.078	102.853	102.452	89.2406	92.4674
s2	1	96.288	100.648	84.9858	92.9497	95.9426	93.2898	89.735	89.0006	78.9186
s3	1.5	75.361	75.2674	78.5719	91.57	82.4555	93.2429	77.1192	85.2267	75.3274
s4	2	72.961	79.8987	84.3376	81.3573	85.2516	74.8672	70.8434	79.0039	79.616
s5	2.5	69.894	75.7815	81.1355	83.0103	83.1875	85.364	66.5618	69.6967	77.1139
s6	3	68.745	66.0003	68.2707	79.9621	88.0008	69.2631	71.6402	66.0852	75.3742
s7	3.5	69.066	71.5029	78.5474	74.8229	73.5829	75.5525	78.834	69.1777	68.973
s8	4	70.482	75.0356	74.1081	70.9764	78.5682	76.7615	70.0291	73.3007	63.7293

## Effect of defects on energy change of rock specimen

In order to explore the influence of defects on rock mass energy, this paper analyzes the energy change between intact rock samples and defective rock samples from the perspective of strain energy. The calculation formula of strain energy in parallel bond model is given in PFC2D.

6 $$\overline{{E_{s} }} = \frac{1}{2}\left( {\frac{{\overline{F}_{n}^{2} }}{{\overline{k}_{n} \overline{A} }} + \frac{{\parallel \overline{F}_{s} \parallel^{2} }}{{\overline{k}_{s} \overline{A} }} + \frac{{\overline{M}_{t}^{2} }}{{\overline{k}_{s} \overline{J} }} + \frac{{\parallel \overline{M}_{b} \parallel^{2} }}{{\overline{k}_{n} \overline{I} }}} \right),$$

where $\overline{{E }_{s}}$ is the strain energy, ${\overline{k} }_{n}$ is the normal stiffness, ${\overline{k} }_{s}$ is the shear stiffness, $\overline{A }$ is the cross-sectional area, $\overline{I }$ is the moment of inertia of the parallel bond cross-section, $\overline{J }$ is the polar moment of inertia of the parallel bond cross-section, ${\overline{F} }_{n}$ is the parallel-bonded normal force, ${\overline{F} }_{s}$ is the parallel-bonded shear force, ${\overline{M} }_{t}$ is the parallel-bonded twisting moment (2D model: ${\overline{M} }_{t}$ = 0) and ${\overline{M} }_{b}$ is the parallel-bonded bending moment.

**Figure 7:**
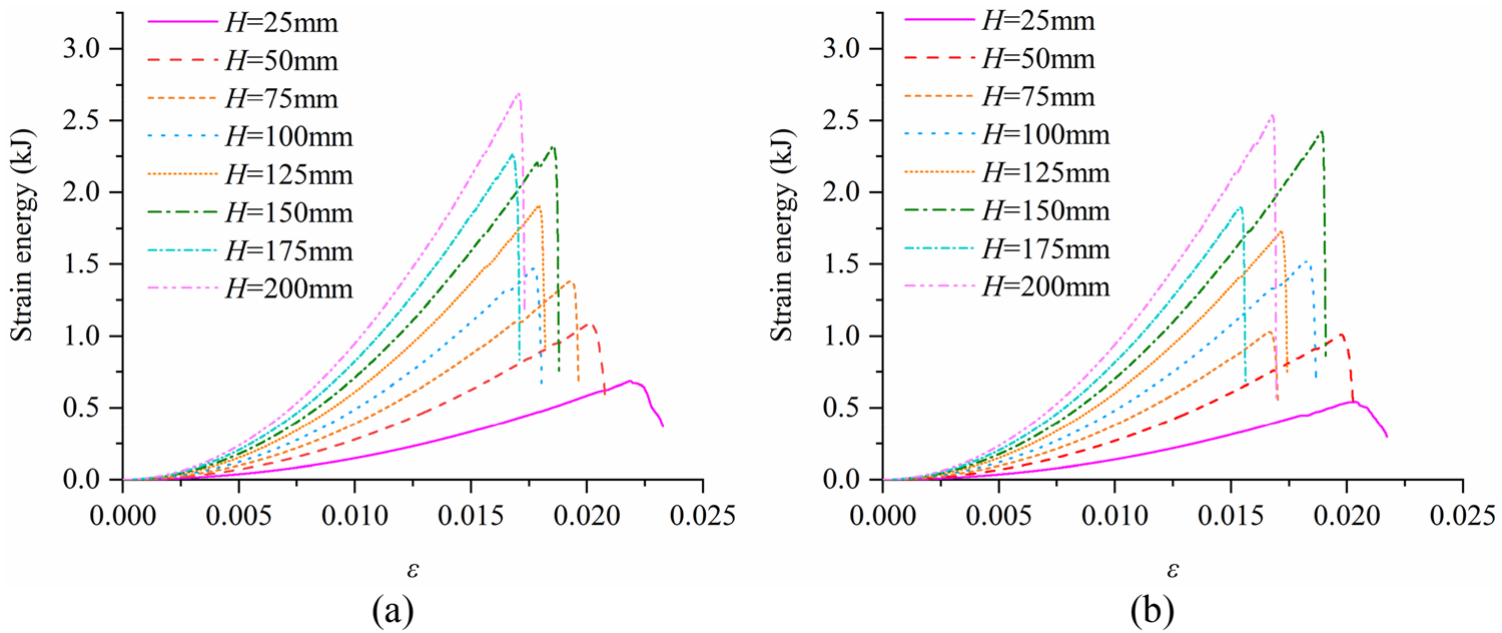
Strain energy curve of rock sample (**a**) Intact rock (**b**) Defective rock.

By recording the change of strain energy during the simulation process, the strain energy curves of samples with different aspect ratios were obtained (Fig. 7). Figure 7 shows the strain energy curves of intact rock samples and defective rock samples at different sizes. It can be seen that the peak strain energy of both defective rock samples and intact rock samples increases with the increase of aspect ratio, and the maximum strain value decreases with the increase of aspect ratio.

**Figure 8:**
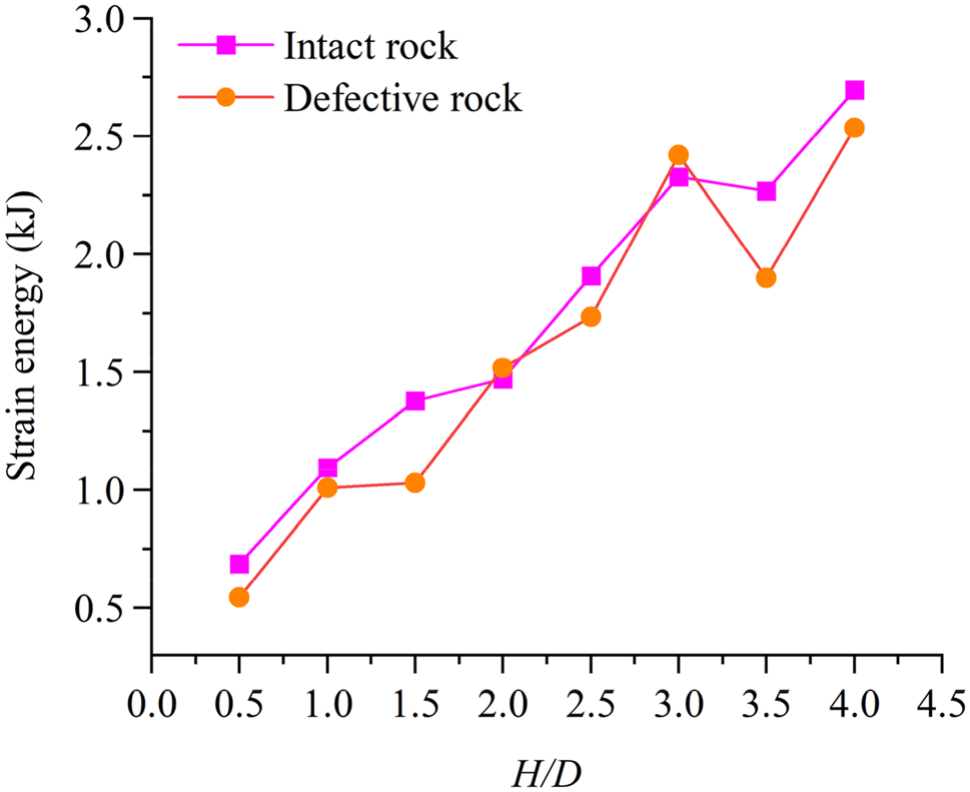
Relationship between strain energy.

Figure 8 depicts the relationship between peak strain energy and aspect ratio. The peak strain energy of the intact rock sample is 0.69 kJ when the aspect ratio is 0.5, and the peak strain energy is 2.69 kJ when the aspect ratio is 4.0, which increases by 2 kJ. The peak strain energy of the defective rock sample is 0.54 kJ when the aspect ratio is 0.5, and the peak strain energy is 2.53 kJ when the aspect ratio is 4.0, which increases by 1.99 kJ. The peak strain energy of the intact rock sample increased by 8.97% on average compared with the defective rock sample. It can be seen that the peak strain energy of the intact rock sample is slightly higher than that of the defective rock sample. And the growth curve of strain energy peak of intact rock sample and defective rock sample is similar.

## Crack characteristics of defective rock specimens

**Figure 9:**
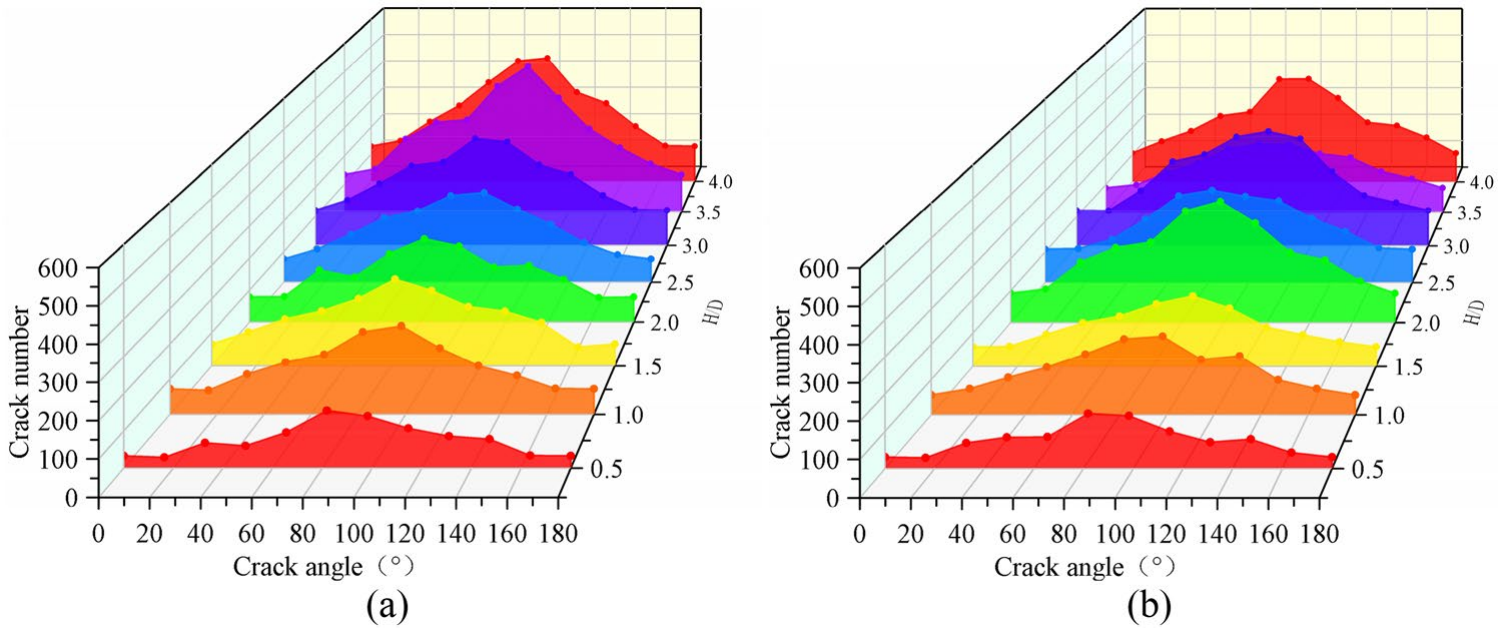
Crack characteristics of intact rock samples crack characteristics of defective rock samples.

Figure 9 depicts the statistical results of the number and angle of cracks in intact rock samples and defective rock samples under different sizes. The number of cracks in intact rock samples and defective rock samples increases with the increase of aspect ratio. And the number of cracks between 65° and 114° is the largest.

**Figure 10:**
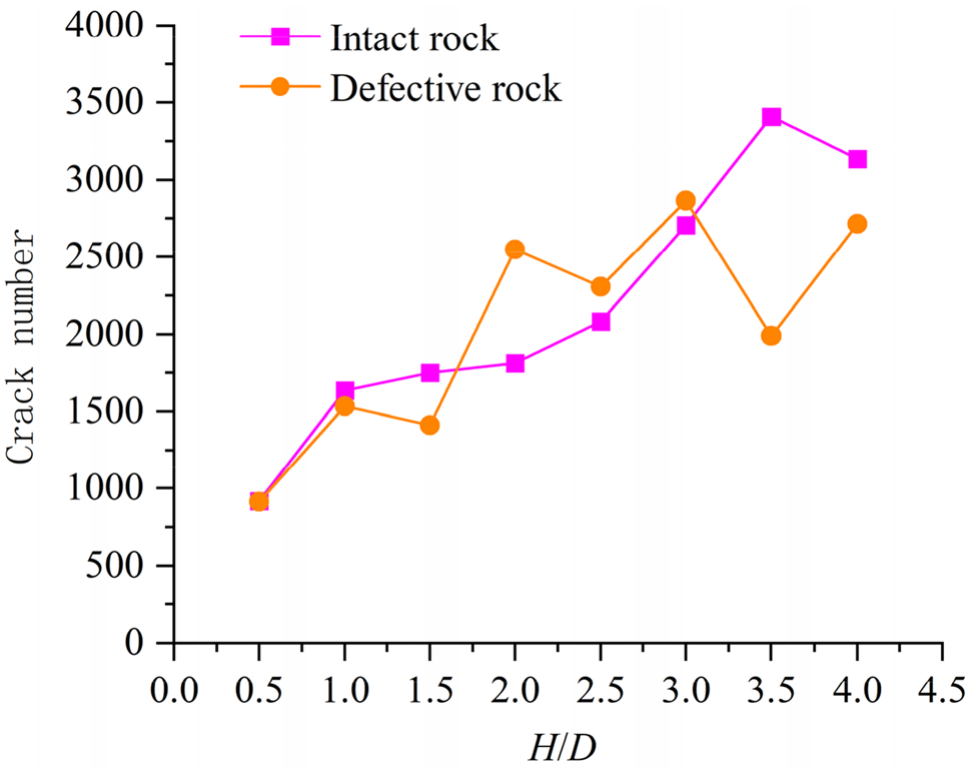
Relationship between crack number and aspect ratio of rock sample.

Figure 10 shows that the number of cracks in intact rock samples and defective rock samples increases with the increase of aspect ratio. However, the number of cracks in intact rock samples is larger than that of defective rock samples. The total number of cracks in intact rock samples is 1176 more than that of defective rock samples, and the growth rate is 7.223%.

## Influence of defects on rock mechanical properties

In order to study the influence of defects on the mechanical properties of rock, this paper analyzes the influence of defects on the failure mode, uniaxial compressive strength, Poisson’s ratio and size effect of rock from the aspects of strength and deformation characteristics of defective rock.

### Effect of defects on deformation characteristics of specimens

In the uniaxial compression test of rock, the deformation characteristics of rock samples are mainly axial compression deformation and lateral expansion deformation after being subjected to uniaxial force. The Poisson’s ratio can well reflect the lateral deformation performance of rock, and the final failure mode can directly reflect the overall deformation of rock sample after failure. In this paper, the influence of defects on the deformation characteristics of rock is analyzed from two aspects of rock failure mode and Poisson’s ratio.

**Figure 11:**
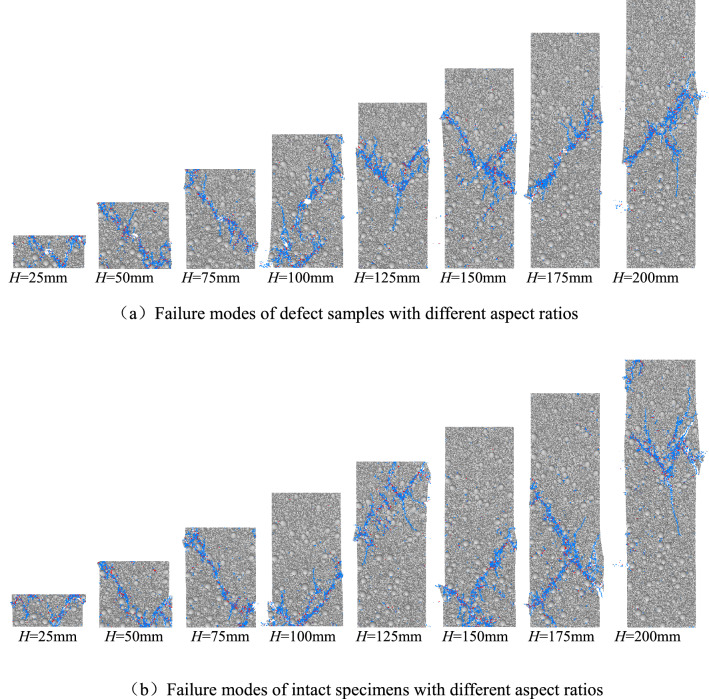
Failure modes of specimens with different aspect ratios.

Figure 11 shows the failure modes of specimens with different aspect ratios after uniaxial compression tests. Groups (a) and (b) are the failure modes of specimens with large defects and intact specimens after uniaxial compression tests. As shown in Fig. 11a, when the aspect ratio of the defect sample is small (*H/D* ≤ 1.5), the failure mode of the defect sample is mainly manifested as the single-slope shear failure through the defect; when the aspect ratio of the defect sample is large (*H/D* > 1.5), the failure mode of the defect sample is mainly manifested as shear failure through the single slope of the defect. As shown in Fig. 11b, the failure mode of the intact sample is mainly manifested as the shear failure of the single inclined plane. Comparing these two groups, it can be concluded that the failure section of the rock sample is greatly affected by the defect position.

**Figure 12:**
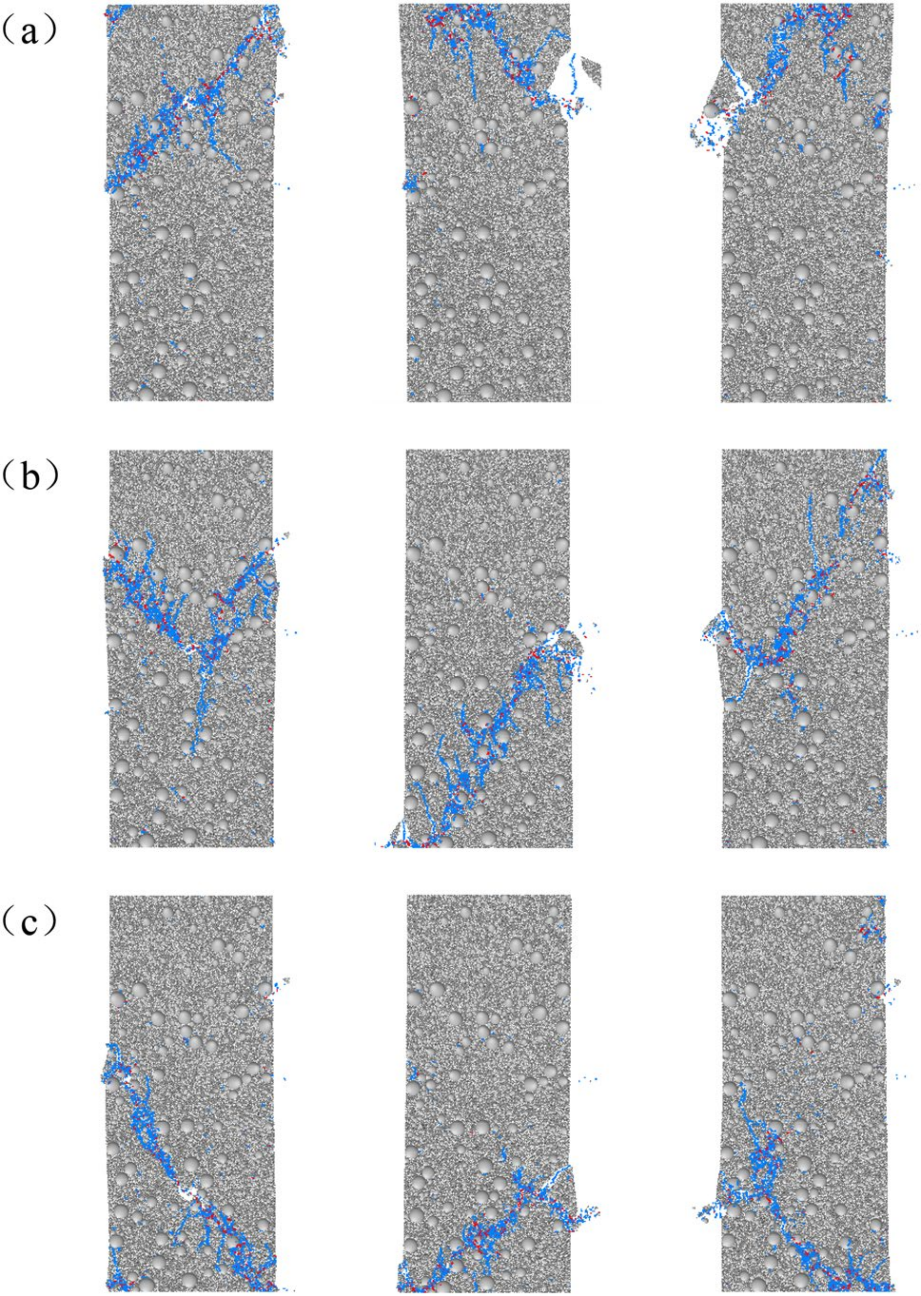
Failure modes of specimens at different defect locations.

As shown in Fig. 12, the failure mode of the same aspect ratio sample after uniaxial compression test is different when the defect position is different. Groups (a), (b) and (c) are the failure modes when the defects are located in the upper, middle and lower parts of the sample. When the defect is in the upper part, the failure mode of the sample is mainly shear failure through the upper end face and the weak surface of the defect (Fig. 12a). When the defect is in the middle, the failure mode of the sample is mainly shear failure through the random weak surface of the defect (Fig. 12b). When the defect is in the lower part, the failure mode of the sample is mainly shear failure through the lower end face and the weak surface of the defect (Fig. 12c). In summary, when the aspect ratio of the sample is constant and the defect position changes, the failure mode of the sample is significantly affected.

**Figure 13:**
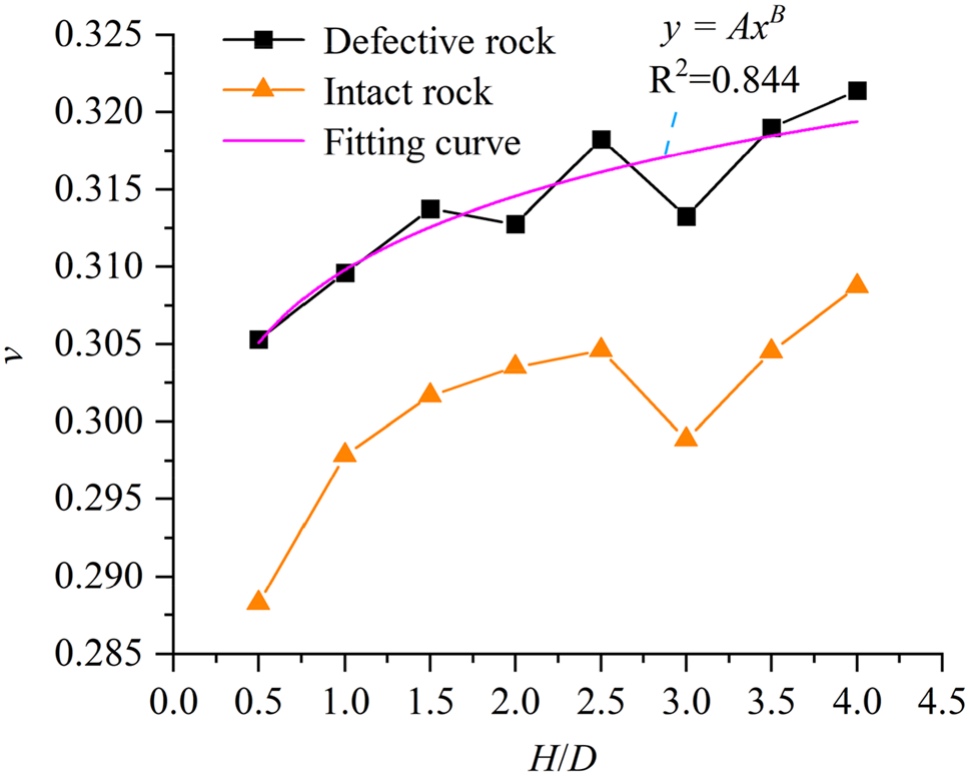
Relationship between Poisson’s ratio and aspect ratio.

In this paper, the displacement change of the sample during uniaxial compression is monitored by measuring the circle and related monitoring commands, and the relationship curve between the aspect ratio and Poisson’s ratio of the intact rock sample and the defective rock sample is obtained (Fig. 13). As shown in Fig. 13, the Poisson’s ratio of the sample fluctuates with the increase of the aspect ratio, but the overall trend is increasing. When the aspect ratio is 0.5, the Poisson’s ratio of the sample is 0.305. When the aspect ratio is 4.0, the Poisson’s ratio of the sample is 0.321, and the growth rate is 5.246%. Comparing the Poisson’s ratio curves of intact rock samples and defective rock samples, it can be seen that the Poisson’s ratio is higher than that of intact rock samples when there are large defects in rock samples, and the average growth value is 0.0132. The relationship between the Poisson’s ratio of the defective rock sample and the aspect ratio of the sample is obtained by fitting the experimental values obtained by the numerical experiment:

7 $$y = Ax^{B} ,$$

where $$A$$ = 0.30979, *B* = 0.02204.

### Effect of defects on strength characteristics of rock specimens

The strength characteristics of rock are characterized by the resistance of rock to maintain its own integrity. In the uniaxial compression experiment, the uniaxial compressive strength of rock is the most intuitive to reflect the strength characteristics of rock. Therefore, this paper starts with the uniaxial compressive strength of rock, and explores the influence of defects on the uniaxial compressive strength of rock samples and the size effect of uniaxial compressive strength.

**Figure 14:**
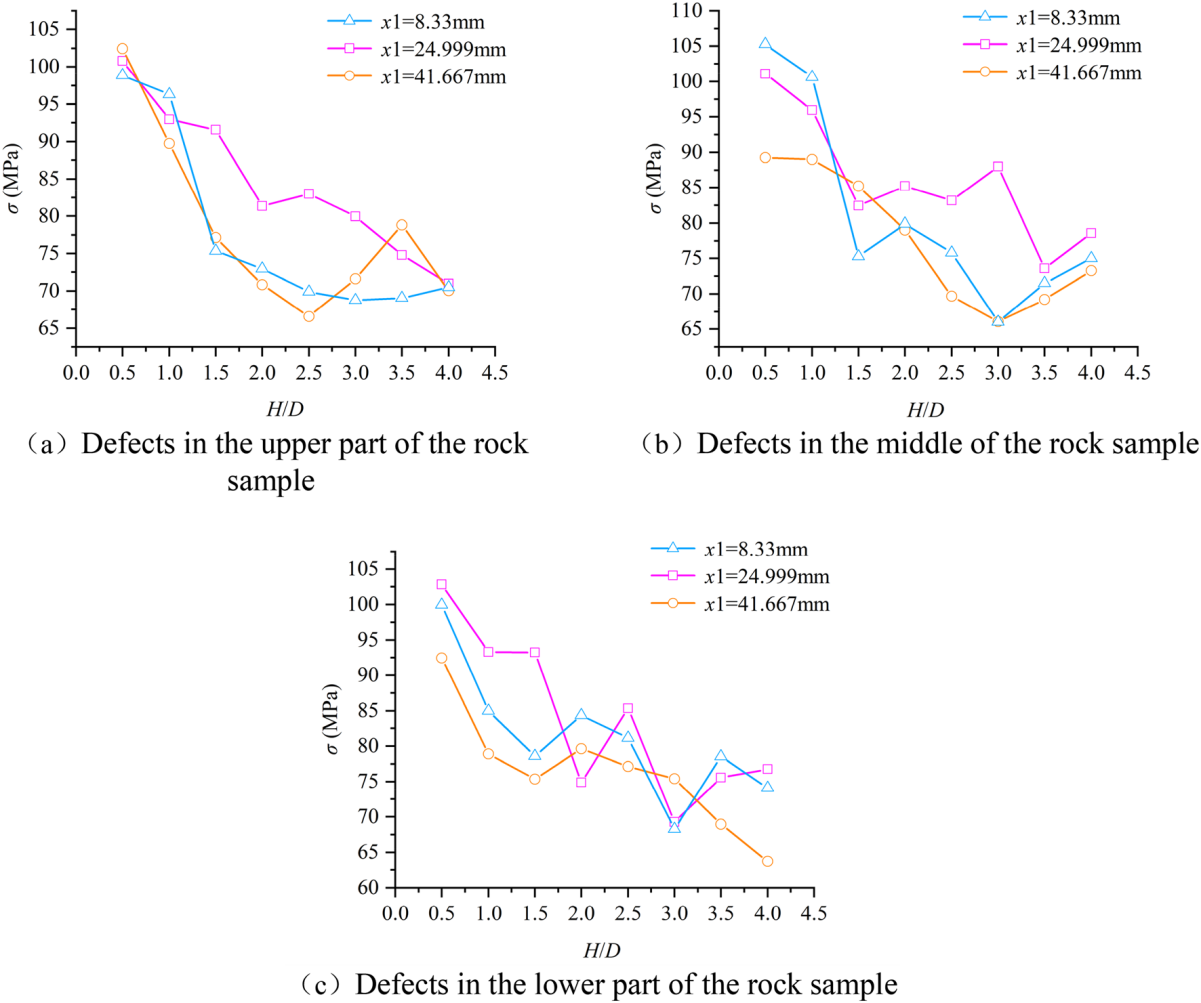
Relationship between uniaxial compressive strength and aspect ratio at different defect locations.

Figure 14 shows the peak strength of specimens with different aspect ratios after uniaxial compression tests at different defect locations. It can be concluded that the location of the defect has a significant effect on the uniaxial experimental results of the rock sample. Therefore, this paper analyzes the relationship between the defect location and the size effect of the rock sample. As shown in Fig. 14a, when the defect is in the upper part, the peak strength of x1 = 24.999 mm (defect in the middle) sample is 7.7% and 8.7% higher than that of x1 = 8.333 mm and x1 = 41.667 mm (defect in the left and right sides) samples. As shown in Fig. 14b, when the defect is in the middle, the peak strength of the x1 = 24.999 mm (defect in the middle) sample is 10.8% and 5.9% higher than that of the x1 = 8.333 mm and x1 = 41.667 mm (defect in the left and right sides) samples. As shown in Fig. 14c, when the defect is in the lower part, the peak strength of x1 = 24.999 mm (defect in the middle) sample is 9.8% and 3.2% higher than that of  x1 = 8.333 mm and x1 = 41.667 mm (defect in the left and right sides) samples. The comprehensive Fig. 14a–c shows that when the defect is located in the middle (x1 = 24.999 mm), the uniaxial compressive strength of the sample is the largest, and the average increase of strength is 7.68%.

**Figure 15:**
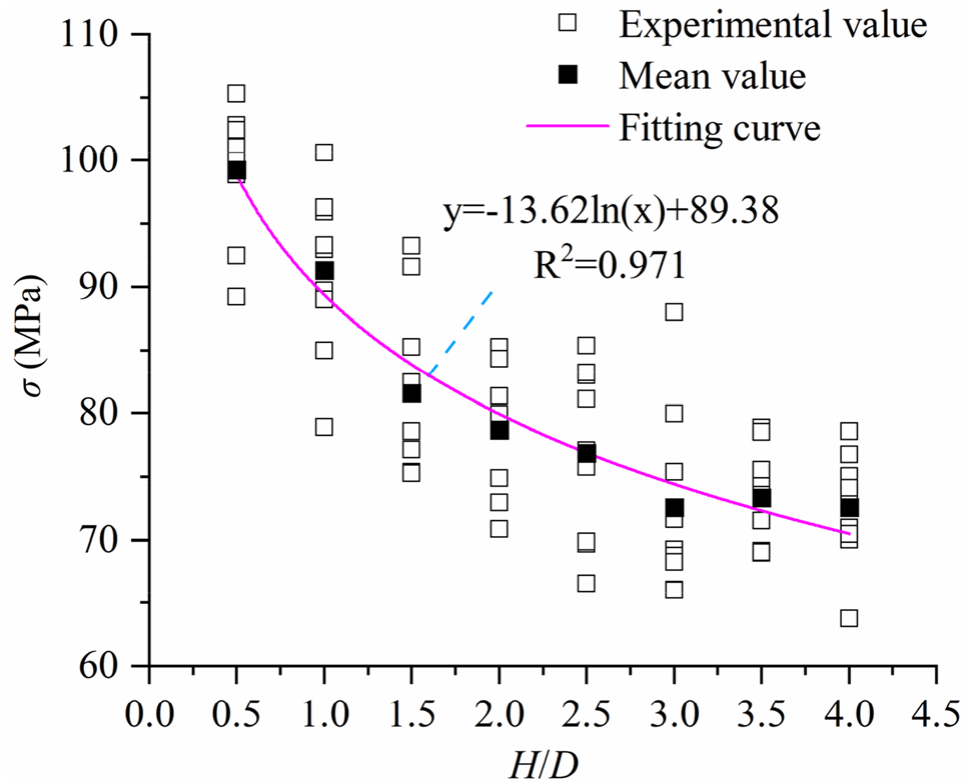
Size effect curve of uniaxial compressive strength of defect specimen.

The uniaxial compressive strength of specimens with defects at different aspect ratios can be obtained from the data in Table 4. The relationship between uniaxial strength and aspect ratio of specimens with defects can be obtained by statistical analysis of uniaxial strength of specimens with defects (Fig. 15). As shown in Fig. 15, the uniaxial compressive strength of the sample with defects also shows a decreasing trend with the increase of the aspect ratio. Fitting the average strength of specimens with the same aspect ratio, the relationship between uniaxial compressive strength and aspect ratio of specimens with defects is obtained:

8 $$y = - 13.62\ln \left( x \right) + 89.38.$$

**Figure 16:**
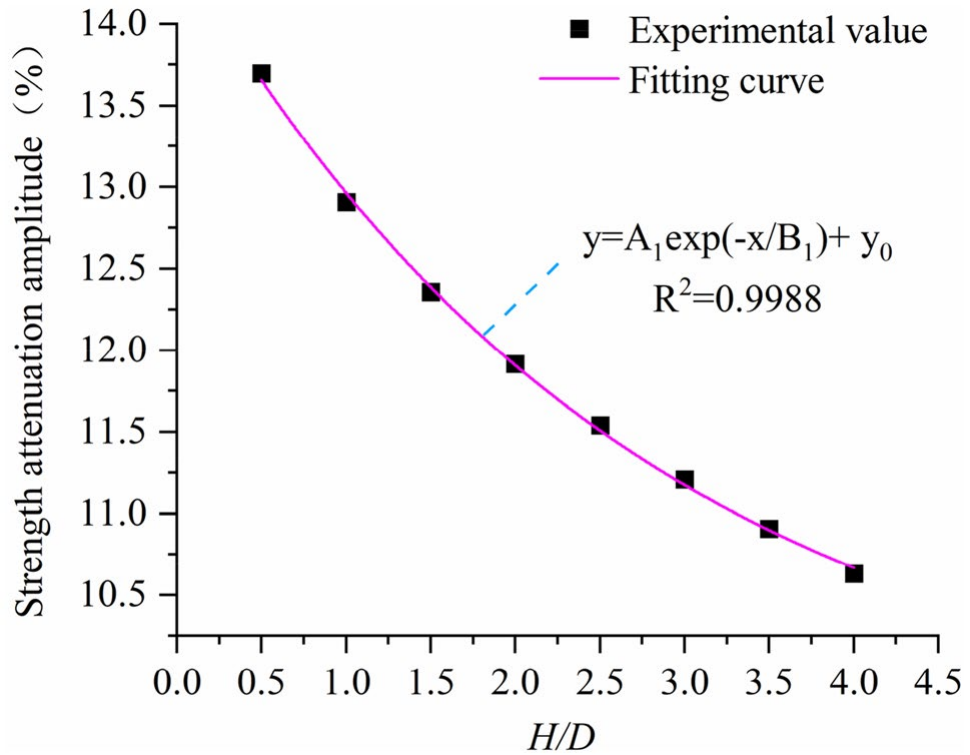
Curve of decreasing range of uniaxial compressive strength.

Comparing the relationship curve between the uniaxial compressive strength and the aspect ratio of the defective sample with the relationship curve between the uniaxial compressive strength and the aspect ratio of the intact rock sample in Fig. 6, it is found that the peak strength of the defective rock sample has a significant attenuation compared with the intact rock sample, and there is also a significant relationship between the attenuation amplitude and the aspect ratio (Fig. 16). As shown in Fig. 16, when the aspect ratio is 0.5, the uniaxial strength attenuation of the sample is 13.70%. When the aspect ratio is 4.0, the uniaxial strength attenuation of the sample is 10.63%. With the increase of aspect ratio, the attenuation amplitude of peak strength of defective rock is also different, and it tends to decrease with the increase of aspect ratio. The relationship between attenuation amplitude and aspect ratio is obtained by fitting the attenuation amplitude value:

9 $$y = A_{1} \exp \left( { - x/B_{1} \;} \right) + y_{0} ,$$

where $${A}_{1}$$ = 0.0497, $${B}_{1}$$ = 2.73205, $${y}_{0}$$ = 0.09518.

From the above fitting formula, it can be concluded that the attenuation range of uniaxial compressive strength of specimens with defects decreases with the increase of aspect ratio, and when the aspect ratio is infinite, the attenuation range is infinitely close to 9.518%.

## Discussions

According to the results of mechanical properties, crack characteristics and energy field, it can be seen that larger size defects have a significant impact on the mechanical properties of rock. Therefore, the influence of defects on the physical and mechanical properties of rock can be obtained by statistical analysis, which provides a reference for the variability of rock experimental parameters and the size effect of rock.

- (1) In the process of rock experiment, energy will be transformed or transferred, and the change of energy can well reflect the overall variation of stress and strain. In the experiment of this paper, the strain energy of rock sample is positively correlated with aspect ratio, and the strain is negatively correlated with aspect ratio. Through the PFC2d program, we try to analyze this phenomenon from a microscopic perspective. As the aspect ratio increases, the size of the sample also increases. Therefore, the strain of the sample will be reduced and the number of bonded bonds between the stressed particles in the sample will also increase. According to the calculation method of strain energy in the parallel bond model introduced above, we can infer that the strain energy required for rock failure increases with the increase of the number of bond bonds. Similarly, when large defects appear in the sample, due to the existence of defects, the number of bond bonds between the particles inside the sample decreases and the failure rate is faster than that of the intact rock sample, so the maximum strain and strain energy will be reduced.
- (2) In the uniaxial compression test of rock, the failure process of rock is: sample compaction (microcrack closure), crack generation, crack propagation, crack growth, sample failure. In this process, the characteristics of cracks can reflect the damage degree of rock mass. In the experiment of this paper, the number of cracks in rock samples is positively correlated with aspect ratio. According to the parallel bond model contact diagram shown in Fig. 1, the number of tensile or compressive shear cracks increases when the contact is in tensile failure or compressive shear failure. The increase of the aspect ratio will lead to the increase of the sample size, so the number of contacts between particles will also increase. This forces the number of cracks that appear when the specimen is destroyed to increase with the aspect ratio. When large defects appear in the specimen, the number of cracks in the rock specimen decreases slightly. Due to the existence of defects, the number of bonding bonds between particles in the sample decreases, so the number of cracks will be reduced.
- (3) The deformation characteristics of rock specimens refer to the characteristics of rock deformation under force. In the uniaxial compression experiment of rock, the deformation characteristics of rock samples are mainly manifested as axial compression deformation and transverse expansion deformation after uniaxial force. Poisson’s ratio can well reflect the lateral deformation performance of rock, and the final failure mode can intuitively reflect the overall deformation of rock samples after failure. In the experiment of this paper, the fracture form of rock sample is obviously affected by the aspect ratio (Fig. 11). As observed in other studies^9^, when the length is small, the failure form of the rock sample is relatively complex, and when the length is large, the rock sample is basically a single section of the single slope shear failure. When large defects appear in the sample, the fracture form of the rock sample is manifested as shear failure through the weak surface of the defect. Due to the occurrence of defects, there is a stress concentration area in the sample, so the stress concentration part is first destroyed during the crack propagation process, and finally the weak surface shear failure through the defect is formed. The Poisson’s ratio of rock samples is also significantly affected by the aspect ratio. As observed in other studies^39^, Poisson’s ratio is proportional to the aspect ratio, that is, as the length of the rock sample increases, the Poisson’s ratio gradually increases. When there are large defects in the sample, the Poisson’s ratio of the rock sample increases. Due to the appearance of defects, the longitudinal normal strain of the sample will decrease, so the Poisson’s ratio value will increase.
- (4) In the uniaxial compression experiment, the most intuitive reflection of the strength characteristics of the rock is the uniaxial compressive strength of the rock. In the experiment of this paper, the uniaxial compressive strength of rock samples is evidently affected by the aspect ratio. When defects appear in the sample, the uniaxial compressive strength of the sample is significantly affected by defects. Due to the appearance of defects, the uniaxial strength of the sample is significantly reduced. When the location of the defect changes, the uniaxial strength of the sample also has a certain impact. When the defects are located on both sides of the specimen, the specimen failure speed is faster and the strength is lower because the weak position is close to the specimen boundary. For the size effect of strength, we found that when there are large defects in the sample, the uniaxial compressive strength of the sample also showed a significant size effect. Because the uniaxial strength of the defect sample is lower than that of the intact rock sample, the defect sample will have strength attenuation under different aspect ratios. Through statistics, it is found that the attenuation amplitude value seems to be affected by the aspect ratio, that is, the strength attenuation amplitude of rock samples with different aspect ratios also changes (Fig. 16). As Zhao Yun et al.^40^ observed, the larger the sample, the greater the number of defects, the greater the uniaxial strength of the sample affected by defects. Because we only set a large defect in this article, and the defect size does not change with the aspect ratio. Therefore, we believe that as the aspect ratio increases, the sample size gradually increases, and the ratio of defect diameter to sample height (*d/H*) gradually decreases, resulting in a decrease in the effect of defects on the sample. In this paper, the aspect ratio of the sample is adjusted by fixing the diameter (*D*) and changing the length (*H*). At the same time, the diameter (*d*) of the defect in the experiment is also fixed, so *D/d* is a fixed value (*D/d* = 10). This paper mainly studies the relationship between the aspect ratio of rock and rock properties. In order to show the relationship between attenuation amplitude and aspect ratio (*D/H*) more directly, the relationship between attenuation amplitude and (*d/H*) can be transformed into the relationship between attenuation amplitude and aspect ratio (10*d/H* = *D/H*).
- (5) Rock mechanics is a science that studies the deformation and failure law of deformed rock mass with the change of environmental stress. In the process of rock formation and long-term geological structure and environmental impact after formation, there are often various types of damage, small cracks and some large voids in the rock. Therefore, we try to study the mechanical properties of defective rock specimens. Due to the preliminary nature of the study, we only performed defect simulation with simple distribution. However, in engineering practice, in addition to the above micro-structural planes (damage, micro-cracks, internal defects, etc.), there are also some discontinuous structures on a large scale. These structures also have a significant impact on the physical and mechanical properties of the rock, resulting in the experimental results can not be accurately predicted. These factors will be considered on the basis of this experiment to do further research.

## Conclusions

In this paper, the numerical model of uniaxial compression experiment is established by particle flow software (PFC2D). On the basis of verifying the rationality of the microscopic parameters of the model, the failure mode, uniaxial compressive strength, Poisson’s ratio and size effect of the rock are analyzed in depth by generating defects at different positions of the sample. The main conclusions are as follows:

- (1) Compared with the complete rock sample, the mechanical properties of the defective rock sample have different degrees of attenuation. From the point of view of energy and crack characteristics, the peak strain energy and total number of cracks of defective rock specimens are slightly lower than those of intact rock specimens. At the same time, the number of cracks and peak strain energy of intact rock and defective rock are positively correlated with aspect ratio.
- (2) The location of defects has a certain influence on the failure mode and uniaxial compressive strength of specimens. The main crack of the specimen is near the end of the defect area according to the defect location. When the defect is in the upper (lower) part, the failure mode of the specimen is mainly shear failure through the upper (lower) end face and the weak face of the defect. When the defect is in the middle, the failure mode of the sample is mainly the random weak surface shear failure through the defect. For uniaxial compressive strength, when the defect is located in the middle of the sample (x1 = 24.999 mm), the average uniaxial compressive strength of the sample is the largest, which is 7.68% higher than that of the defect on the left and right sides (x1 = 8.333 mm, x 1 = 41.667 mm ).
- (3) The Poisson’s ratio of intact rock and defective rock increases with the increase of aspect ratio. Through the fitting analysis of this growth trend, the relationship formula between Poisson’s ratio and aspect ratio is obtained. Comparing the defective rock samples with the intact rock samples, it is found that the Poisson’s ratio of the defective rock samples is generally higher than that of the intact rock samples, and the average growth is 0.0132.
- (4) When there are large defects in the sample, the uniaxial compressive strength of the sample also shows obvious size effect. By comparing the relationship between the uniaxial compressive strength and the aspect ratio of the intact sample and the defective sample, the attenuation amplitude value of the defective sample relative to the intact rock sample is obtained. Through analysis, it is found that the attenuation amplitude value also shows a decreasing trend with the increase of the aspect ratio, and the relationship formula between the attenuation amplitude and the aspect ratio is fitted.

## Data Availability

The datasets used and/or analysed during the current study available from the corresponding author on reasonable request.
